# Bone mineralization and immediate function of six dental implants in patients with Klinefelter syndrome

**DOI:** 10.1002/ccr3.3142

**Published:** 2021-10-04

**Authors:** Galina Ciobanu, Francesca De Angelis, Eugen Slabari, Giorgio Pompa

**Affiliations:** ^1^ Department of Stomatological and Maxillofacial Surgery oral implantology "Arsenie Gutan" Histology Unit University N. Testemitanu University of Medicine and Pharmacy of Moldova Chisinau Moldova; ^2^ Prosthodontics Unit Department of Odontostomatological and Maxillofacial Sciences “Sapienza,” University of Rome Rome Italy

**Keywords:** bone mineralization, immediate loading, Klinefelter

## Abstract

Patients with Klinefelter syndrome face many challenges in oral treatment and bone mineralization due to multiple systemic dysfunctions. This case report follows the geometrical treatment with immediate implant loading of an adult male patient with Klinefelter syndrome. Satisfactory results were demonstrated in clinical follow‐up.

## Graphical abstract

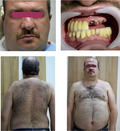

## 1 BACKGROUND

Certain metabolic diseases, including Klinefelter syndrome, cause severe atrophy in the upper and lower jaw of patients, primarily males. The question then arises whether or not immediate implant loading can be performed in a single day. Due to complications with bone mineralization and implant osseointegration, and further complicated by the abnormal abundance of trabecular bone and lacuna in the maxilla, the long‐term prognosis of KS patients requiring immediate implant loading in the upper jaw is limited.

The chromosomal abnormality of Klinefelter syndrome, KS, is characterized in 80% of cases by 47, XXY, and in 20% by the XXXY karyotype. These genes influence the hypothalamus. The hypothalamus directs the entire endocrine system and is the most important determiner of hormones in the body. While a diagnosis of KS is often made in childhood as the result of observed behavioral problems or learning disabilities, if left undiagnosed, it will cause significant hormonal problems and interfere with the sexual maturation of patients. Untreated KS can cause dramatic physical effects and is responsible for increased morbidity and decreased life expectancy. Congenital malformations of the heart, as well as malformations in the urinary, muscular, and endocrine systems, result in hypogonadism, diabetes, and hypothyroidism. Furthermore, the low levels of testosterone associated with KS increase the risk of vascular diseases and diabetes independent of chromosomal factors.^1^ Mental illnesses associated with KS also contribute to increased morbidity.^2^

A characteristic facial morphology of individuals with KS is mandibular prognathism. A study on KS subjects showed increased flexion of the cranial base, with a greater genial angle and pronounced jaw. A shortening of the anterior cranial base has also been noted, as have abnormalities in facial height, the mandibular branch, and special dimensions of the dental crown.^3^

The bodies of KS patients are characterized by increased fat mass and decreased lean body mass. Reduced bone mass has likewise been found in up to 48% of cases.^4^ Patients with KS also have a testosterone deficiency resulting in early osteoporosis and insufficient insulin‐like factor 3 (INSL3).^5^ Reduced bone mass, combined with low testosterone levels, results in a bone mineral deficit and causes an abundance of lacuna in the bone, a major obstacle for successfully performing oral surgeries.

KS patients have a higher prevalence of class I caries. KS patients therefore tend to lose their teeth at an early age. Congenital enamel deficiency (weak, soft enamel) contributes to this early tooth loss, as do the problems with hygiene associated with the mental effects of KS. Furthermore, the regular medications taken by some patients, such as tranquilizers and anticoagulants, reduce saliva secretion, which in turn promotes the onset of caries, and, ultimately, complete tooth loss. Moreover, early tooth loss is exacerbated by the gum inflammation and periodontal diseases which accompany hormonal imbalances.^6^

The purpose of the present study is to evaluate several topics relating to Klinefelter syndrome, KS. The first is the reaction of the metabolic system to bone remodeling under high compressive stress. The stress patterns were induced by conventionally placed and tilted implants supporting a fixed prosthesis in a patient with Klinefelter syndrome. The second is to better understand the hormonal stress on bone in KS patients. And the last is to evaluate dental implant placement practices.

This case report, based on a critical discussion of available results, proposes clinical and practical recommendations for KS screening and management in order to detect, prevent, or treat the decline in bone mass in KS patients using immediate dental implant loading.^7^

### 1.1 Case presentation

This patient's preference for fixed implant restoration is the inclusion criteria for this study. The patient is a 38‐year‐old man suffering from Klinefelter syndrome, KS. Due to this syndrome, the patient suffers from other systemic cardiovascular diseases, hormonal imbalances, and mental disorders. The mental disorders are especially important to consider because they interfere with the patient's ability to follow care instructions, and thus elevate the risk of dental implant loss or bone loss.

The patient had an atrophic full edentulous maxilla. However, the patient was not willing to undergo bone augmentation procedures and instead required a dental implant. Compared to normal patients, the subject of our case has an abundance of trabecular bone with a large amount of enlarged lacuna. In this case, it is imperative to stimulate the bone. This, combined with the patient's inability to follow care instructions, prompted the decision to perform the entire procedure in a single day using immediate dental implant loading.

### 1.2 Treatment

#### 1.2.1 Surgical procedures

**FIGURE 1:**
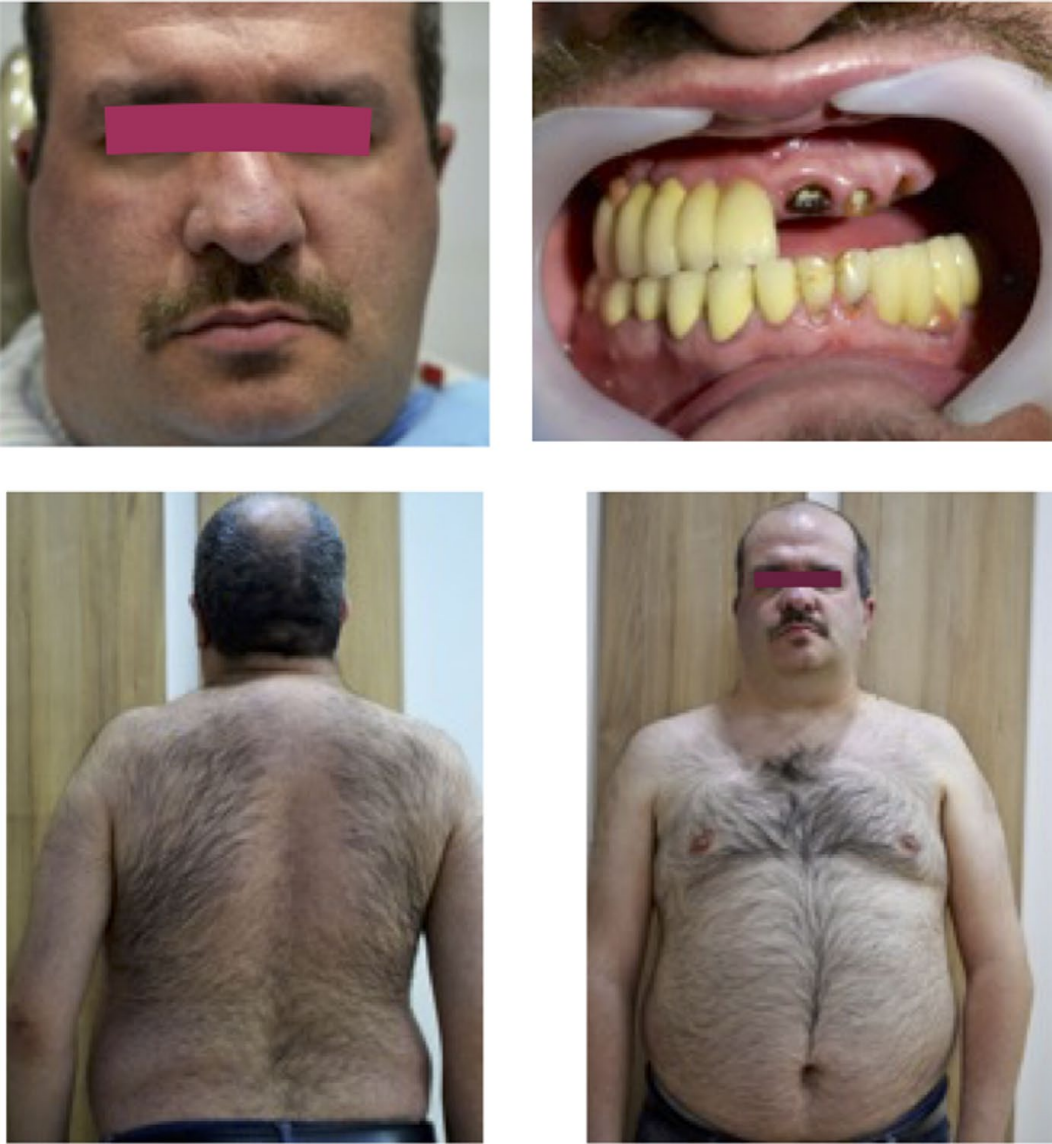
Hypogonadal patient, Klinefelter syndrome before surgery

The 3D geometry of the maxilla, consisting of cortical and trabecular bone, was reconstructed with a computerized tomography scan (Figure 1).

**FIGURE 2:**
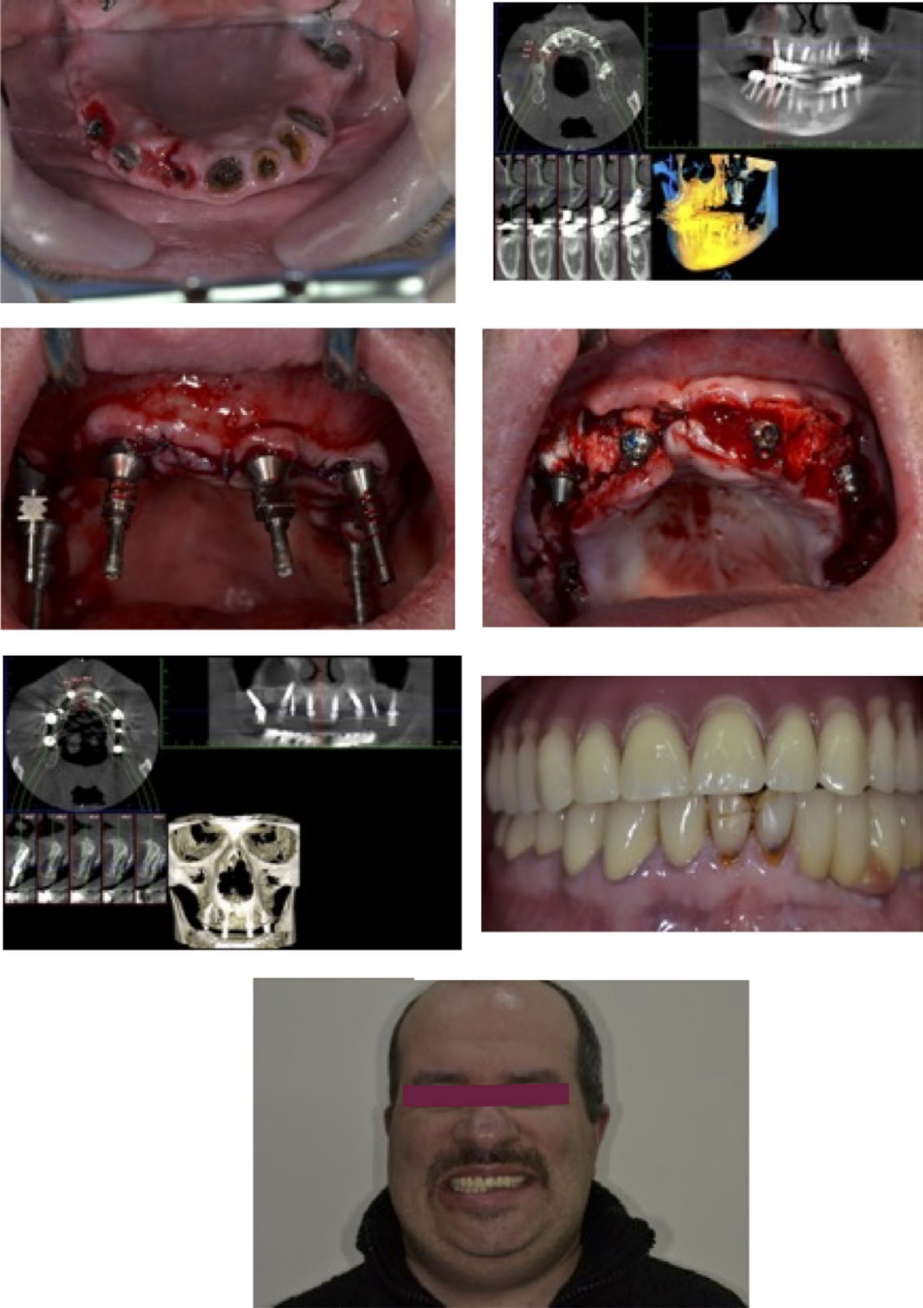
Surgical protocol treatment before and after 3D planing. Immediate loading

After administration of local anesthesia with 2% mepivacaine with 1:100 000 epinephrine (France), a midcrestal incision was made from the pterygoid area. A mucoperiosteal flap was elevated and all roots with granuloma were removed, as were implants with peri‐implantitis. All parts of the bone were irrigated. The maxillary sinus walls were not touched. The only zones disturbed were as follows: (a) the lateral incisor area with a zero degree implant, (b) the premolar‐canine implant at 35 degrees, and (c) the molar II‐molar III implant at 17 degrees. The patient received six implants with the same protocols, and implant angulation was determined using the triangle of bone concept. The measurements were performed in both the buccal‐oral and mesio‐distal directions and repeated twice for every direction. The mean of the four measurements was then calculated. After a healing period of 3‐6 months, the prosthetic rehabilitation phase could be started (Figure 2). Multiunit abutments were connected to the implants, and an impression was taken. If the final torque of any implant was under 50 Ncm, cover screws were connected (30 Ncm were needed in zone I and 17 Ncm in zones II and III) and prosthetic fabrication was delayed for one hour, after which all crown copings were fixed with 15 Ncm.

Bone densitometry was performed following the procedure using computer tomography. 3D scanning was used to evaluate the soft and hard tissues, the maxilla bone anterior, the maxilla bone posterior, and the maxilla pterygoid bone area (Table 1). Implants were evaluated using ISQ: implant anterior—0 degrees; posterior—35 degrees; and pterygoid‐maxilla area—17 degrees. All implants were measured with Osstell ISQ at the time of the immediate loading and every 3 months after for 1 year (Table 2).

**TABLE 1:** Geometrical marginal bone loss (in millimeters) according to implant location of KS patient

	Before surgery KS soft‐hard tissues	After surgery KS soft‐hard tissues	6‐mo KS soft‐hard tissues	1‐y KS soft‐hard tissues	*P*
CT, zone I, straight	m‐11.2	m‐11.2	11.2 ± 0.2	11.5 ± 0.4	.42
CT, zone II, tilted	m‐14.4 d‐16.4	m‐14.4 d‐16.4	m‐14.4 ± 0.2 d‐16.4	m‐14.4 ± 0.4 d‐16.4 ± 0.3	0
CT zone III, tilted	m‐13.8 d‐11.1	m‐13.8 d‐11.1	m‐13.8 ± 0.2 d‐11.14 ± 0.2	m‐13.8 ± 0.2 d‐11.14 ± 0.4	0

Note

Zone I—included zone incisive and canine; Zone II—premolar area and I molar; Zone III—included II molar and III molar aria. m—mesial implant; d—distal implant.

**TABLE 2:** Periotest values (implant stability) on the ISQ scale for a KS patient at implant placement, 6‐mo, and 1‐y follow‐up

ISQ	KS—immediate placement ISQ	KS—6‐month ISQ	KS—1‐year ISQ	*P*‐value
Maxilla, anterior Zone I, 0*	45	50	80	.41
Maxilla, posterior Zone II, 35*	19	50	80	.2
Maxilla, pterygoid Zone III, 17*	30	65	80	.11

Note

To monitor osseointegration, measuring at placement and before final restoration.

Abbreviation: SD: standard deviation (*t* test).

Prosthetic protocol: The patient's maxilla had atrophied. The patient also appeared to have an Angle Class III malocclusion. Irreversible hydrocolloid impressions were taken to obtain two casts from the residual ridge of the patient before surgery. These casts were used to mold open custom trays and record base waxes.

The fixed prosthetic was prepared one day before surgery. After the procedure, another impression was taken in order to make precise adjustments. The crowns of the temporary prosthetic are fixed to the coping with adhesive directly in the oral cavity.

For immediate loading, a 35‐degree angled multiunit abutment, a 17‐degree angled multiunit abutment in the posterior implant, and a straight multiunit abutment for the anterior implant were connected to the prosthesis. All straight implants were torqued 30 Ncm, and the angulated abutments were torqued 20 Ncm according to the manufacturer's recommendation. A prosthetic temporary tooth was fixed directly to the coping in the oral cavity with resin. Occlusion was adjusted, and mutually protected occlusion was established. The patient was placed on a soft diet.

Prosthetic procedures were carried out by a prosthodontist. Baseline radiographs were taken on the day of delivery, since it is inconvenient for patients with a severely resorbed mandible to hold integral films in the correct position. Panoramic radiographs, TC, were taken in order to evaluate the marginal bone level. The patient came to the clinic every week for cleaning for 3 months following the procedure. In the absence of pain or any other complications, follow‐up visits were scheduled at six‐ and twelve‐month intervals. After that, yearly visits were established in order to monitor prescriptions, CBCT, blood analyses, and hormonal levels.

## 2 RESULTS

The implants appear stable, with no suppuration or pain at the implant site. The prosthesis is considered stable if it functions without pain or mobility. Marginal bone levels were recorded using radiographs calibrated by the length of each implant. CT radiographs were digitalized and imported to Romexis R version 2.6 software (Planmeca, IL, USA). Marginal bone loss was defined as the distance between the implant shoulder and the first bone‐implant contact, using the medial and distal aspect of each implant in millimeters. The mean value of medial and distal bone loss of each implant was insignificant. The implants were stable, and there was no implant loss.

Blood samples were evaluated before surgery and then every 3 months postsurgery for 1 year. Absolute data were expressed as the mean + one standard deviation (SD) of the mean. Comparisons between groups were performed by a Student's t test for continuous data after the acceptance of normality of the data. *P*‐values (two‐sided) <.05 were considered as statistically significant.

The cardiovascular risk factors (CRFs) of the subject are a waist circumference >102 cm, hypertension (blood pressure >130/85 mm Hg found in three different measurements), HDL cholesterol <40 mg/dL, triglycerides >150 mg/dL, LDL cholesterol >160 mg/dL, and a homeostasis model assessment (HOMA) >2.4. The subject is also a smoker. (Table 3).

**TABLE 3:** Biochemical, hormonal, and some clinical data of KS patient in 3‐, 6‐, and 9‐mo follow‐up

	Klinefelter	Klinefelter	Klinefelter	Klinefelter	*P*‐value
	Before surgery	3 mo	6 mo	9 mo	
Testosterone (nmol/L)	11	10	11	11	.001
Estradiol (pmol/L)	113	113	110	109	.001
FSH (IU/L)	31.0	30,2	32	31,1	.001
T4 nmol/L	94,5	94,3	90,5	92,6	.003
TSH mLU/	0,58	0,48	1,47	0,98	.0012
HDL cholesterol (mg/dL)	47	48	45	47	0.318
Triglycerides (mg/dL)	146	148	147	145	.0123
Body mass index (BMI)	26	24	24	25	.0479
Waist (cm)	98	98	95	96	.391
GluC mmol/L	6.6	6.6	6.6	6.6	.001
Hb1C%	5,9	6	6	5,8	.001
INR	1,49	1,49	1,49	1,49	0.001

We found that a patient with KS had significantly higher levels of T3, PTH, and estradiol, as well as lower levels of testosterone and EPCs than the control's INR (Table 3).

Postsurgical blood and hormonal analysis showed a correlation between hormonal levels, bone mineralization, and osseointegration of the implant. This correlation deserves further study (Table 2).

It is possible, however, that the differences found between KS studies might be related to the bone site being examined, given the varying proportions of trabecular and cortical bone found at specific sites on the skeleton and the higher sensitivity of trabecular bone to testosterone (Figure 1).

### 2.1 Discussion

The aim of the present study was to examine the immediate implant loading rehabilitation of edentulous jaws with four tilted implants and two straight implants in a patient with KS.^8^

In standard practice, KS patients receive an immediate provisional acrylic prosthesis, but this prosthetic usually breaks. For such patients, it is more advantageous to fix the coping to the crown in the oral cavity.^9^ A high retention rate of immediate loading has been reported for healthy patients in the literature, but this is not the case for patients with a hormonal imbalance as found in KS.^10^, ^11^

Single‐day geometrical placement of abutments and prosthetic teeth in KS patients provides a great opportunity for osseointegration without mental stress. Stress is an important factor to consider because stress blocks the hormones essential for osseointegration. Reduced pain is another factor as pain also acts as a hormone blocker.

The findings show that T levels below 200 ng/dL result in a significant decrease in bone mineral content while KS patients with normal T levels have normal bone content.^12^ The patient in this study had normal T levels.

T cells are responsible for intercellular signaling and detecting mechanical pressure and loads. This mechanism functions to help adapt bone to mechanical forces.^13^ Osteocytes seem to coordinate the bone remodeling process by regulating both osteoclast and osteoblast activity. Bone lining cells, osteocytes, cover surfaces where there is no bone resorption or formation, but they play a role in preventing local bone resorption by inhibiting osteoclasts.^13^

In adulthood, estrogen helps maintain bone mass by inhibiting osteoclastic bone resorption. While testosterone influences trabecular bone, estrogen receptors are primarily found in cortical bone. This means cortical bone is most sensitive to estrogen changes which inhibit osteoclastic bone resorption.^14^

The effect of T cell–stimulating hormones, which in turn stimulates increased levels of bone metabolism, was found to be due to unregulated gene expression in KS patients. This significantly influences osteoclastic function, and KS patients demonstrate bone resorption similar to that of postmenopausal women. KS patients therefore essentially suffer the effects of osteoporosis.^15^

Several studies have been conducted to determine whether a decrease in bone formation or an increase in bone resorption is present in KS. There is a significant reduction in the bone formation markers osteocalcin and bone‐specific alkaline phosphatase (BSAP) in both young and adult untreated KS patients.^16^ However, contradictory results on bone markers in KS have been published.^17^, ^18^ Differences in populations, such as age, the duration of testosterone supplementation, the degree of obesity, and the mental state of the patient, are all possible explanations for these differences in bone markers.^19^ Whereas both mechanisms of bone formation and resorption seem to be present in adult KS patients, a study on children and adolescents with KS noted only a reduction in bone markers.^20^ Prospective and interventional investigations are needed to define if increased follicle‐stimulating hormone (FSH) production in hypogonadal men is related to a decrease in bone density. It also needs to be determined whether this effect is indeed attributable to the stimulation of osteoclastic bone resorption.^21^

In many studies, KS patients tend to have lower 25 OH vitamin D levels than healthy subjects. The widespread recent interest in the ability of vitamin D to prevent bone fractures has also generated studies evaluating vitamin D and its effect on the bone composition of KS men. Undiscovered X chromosomal factors, a higher degree of adiposity, and lowered sunlight exposure may all contribute to a lower vitamin D status in KS, but these have not been investigated in previous studies and the exact cause of this deficiency remains unknown.^22^, ^23^ The impaired bone mineralization around dental implants in male KS patients is probably related to several factors including hypotestosteronemia, vitamin D deficiencies, and decreased physical activity and muscle strength.^24^ Giving our patient vitamins D and B improved both the bone mineralization and bone turnover in the patient. These vitamins stimulate the intestinal absorption of calcium and phosphate and are therefore important for bone mineralization.

Most research into immediate loading dental implants is only about healthy patients. No information is available regarding patients with metabolic diseases, nor are there recommendations for treatment. In our case, testosterone levels were so low that it was unnecessary to determine the level of hypotestosteronemia in order to indicate the necessity of testosterone therapy. The same is true for determining the duration and intensity of physical activity needed in order to maintain normal bone mineral density (BMD) in late adolescence or young adulthood when interventions will be most effective. Previous studies have reported that incidences of mechanical complication are higher when a bridge contains a mix of implants and teeth.^25^ This complication was not a factor in this case as the patient's teeth were already severely damaged. Regardless, fixing a bridge containing only implants is recommended.

KS is associated with implant tooth loss, as well as significant morbidity due to vascular diseases and osteoporosis. Several factors, such as low hormonal status, vitamin D and B deficiencies, lower muscle strength, decreased activity, and impaired mentality, appear to negatively influence the process of bone mineralization in KS. Given the lack of well‐performed prospective studies, evidence‐based preventive actions and optimal therapeutic interventions are not yet possible.

## 3 CONCLUSION

The present report shows that if a surgeon performs geometrical 3D planning and a biomechanical crown treatment is combined with a hormonal system in equilibrium, a great result can be obtained without mechanical or systemic complications.

## CONFLICT OF INTEREST

None declared.
